# Predicting Long-Term Health-Related Quality of Life after Bariatric Surgery Using a Conventional Neural Network: A Study Based on the Scandinavian Obesity Surgery Registry

**DOI:** 10.3390/jcm8122149

**Published:** 2019-12-05

**Authors:** Yang Cao, Mustafa Raoof, Scott Montgomery, Johan Ottosson, Ingmar Näslund

**Affiliations:** 1 Clinical Epidemiology and Biostatistics, School of Medical Sciences, Örebro University, 70182 Örebro, Sweden; scott.montgomery@oru.se; 2 Department of Surgery, Faculty of Medicine and Health, Örebro University, 70182 Örebro, Sweden; mustafa.raoof@regionorebrolan.se (M.R.); johan.ottosson@regionorebrolan.se (J.O.); ingmar.naslund@regionorebrolan.se (I.N.); 3 Clinical Epidemiology Division, Department of Medicine, Karolinska Institutet, 17177 Stockholm, Sweden; 4 Department of Epidemiology and Public Health, University College London, London WC1E 6BT, UK

**Keywords:** prediction, deep learning, conventional neural network, health-related quality of life, bariatric surgery

## Abstract

Severe obesity has been associated with numerous comorbidities and reduced health-related quality of life (HRQoL). Although many studies have reported changes in HRQoL after bariatric surgery, few were long-term prospective studies. We examined the performance of the convolution neural network (CNN) for predicting 5-year HRQoL after bariatric surgery based on the available preoperative information from the Scandinavian Obesity Surgery Registry (SOReg). CNN was used to predict the 5-year HRQoL after bariatric surgery in a training dataset and evaluated in a test dataset. In general, performance of the CNN model (measured as mean squared error, MSE) increased with more convolution layer filters, computation units, and epochs, and decreased with a larger batch size. The CNN model showed an overwhelming advantage in predicting all the HRQoL measures. The MSEs of the CNN model for training data were 8% to 80% smaller than those of the linear regression model. When the models were evaluated using the test data, the CNN model performed better than the linear regression model. However, the issue of overfitting was apparent in the CNN model. We concluded that the performance of the CNN is better than the traditional multivariate linear regression model in predicting long-term HRQoL after bariatric surgery; however, the overfitting issue needs to be mitigated using more features or more patients to train the model.

## 1. Introduction

Severe obesity, defined as having a body mass index (BMI) greater than 40 kg/m^2^ or greater than 35 kg/m^2^ plus at least one obesity-related comorbidity [1,2], has been associated with numerous health outcomes and reduced health-related quality of life (HRQoL) [3,4,5,6,7,8]. HRQoL measures population health multi-dimensionally from physical, mental, emotional, and social functioning domains, which have already been identified as an important indication for bariatric surgery and recognized by the United States National Institutes of Health Conference as early as 1991 [9,10]. Although many studies have reported changes in HROoL after bariatric surgery, few are long-term prospective studies. A systematic review of seven prospective cohort studies with a follow-up time of ≥5 years revealed that bariatric surgery patients reported considerably improved HRQoL and the improvement was maintained over the long term [11]. However, many patients still experience reduced HRQoL after surgery. In our study, 39% of patients had significant improvements in physical functioning (PF) (increased by >25 in the original score or >0.25 in the scaled score), and the rest had no significant improvement and some patients (2%) even had significant deterioration (reduced by >25 in the original score or >0.25 in the scaled score). No relationship between the PF scores before and 5 years after surgery was identified (Figure S1).

Although some preoperative psychological factors, including personality change, severe psychiatric disorder, or depressive symptoms, are associated with postoperative HRQoL after bariatric surgery [12,13], whether long-term HRQoL after bariatric surgery can be predicted based on patients’ baseline features has not been investigated. The present study examined the performance of the convolution neural network (CNN) for predicting 5-year HRQoL after bariatric surgery based on the available preoperative information from a national quality registry, and compared CNN with a conventional linear regression estimator.

## 2. Material and Methods

### 2.1. Patients and Features

Data for the patients registered in the Scandinavian Obesity Surgery Registry (SOReg) were used for the current study. The SOReg was launched in 2007 and covers 98% of bariatric surgery in Sweden since 2009. SOReg is validated regularly and has been shown to have high data quality [14]. In total, 27 - of 42 operating centers in Sweden participate in the HRQoL registration in SOReg. HRQoL was measured using the RAND-SF-36 and the obesity-related problems (OP) scale preoperatively and 1, 2, and 5 years after surgery. In the present study, preoperative and 5-year HRQoL data, including PF, role physical (RP), bodily pain (BP), general health (GH), vitality (VT), social functioning (SF), role emotional (RE), mental health (MH) scale, summary physical scale (PCS), summary mental scale (MCS), and OP, were used. All scale scores ranged from 0 to 100, with higher scores indicating better health status except for OP, where low values represent good health. Eight baseline features, including sex, age, BMI, sleep apnea syndrome (SAS), hypertension, diabetes, dyslipidemia, and depression, were also used as predictors.

In total, 6687 patients with complete information on 19 baseline features and 11 5-year HRQoL measures were used in the machine learning study.

The data that support the study are not publicly available because they contain information that could compromise research participant privacy and confidentiality. The authors will make the data available upon reasonable request and with permission of the Committee of Scandinavian Obesity Surgery Registry in Örebro, Sweden.

### 2.2. Feature Scaling

Before machine learning, the features in the dataset were scaled. The binary features were converted into dummy variables, and the continuous features were scaled to between 0 and 1 using a min-max scaler. In the sensitivity analysis, the normalizer and standardizer scalers were also used to evaluate the influence of scalers on the model’s performance.

### 2.3. Conventional Neural Network

A CNN is a regularized version of a multi-layer perceptron neural network, which was inspired by a biological process where the connectivity pattern between neurons resembles the organization of the visual cortex [15]. Although not specifically developed for non-image data, CNN may achieve state-of-the-art results on regression prediction problems, especially for data with time series or spatial patterns. The CNN input is traditionally two-dimensional (2D) but can also be changed to be one-dimensional (1D), allowing it to develop an internal representation of a 1D sequence. In our study, we used a CNN with seven hidden layers, including two 1D convolution layers (with 10 filters for each), two 1D max pooling layers, one flattened layer, and two dense layers (with 1000 computation units). The rectified linear unit (relu) activation function was used for the convolution layers and dense layers, and the normal distribution was used to initialize weights in the layers. The mean squared error was used as the loss function and the an Adadelta algorithm was used as optimizer when compiling the model [16]. The structure of the CNN model is shown in Figure S2.

### 2.4. Model Validation and Evaluation

In total, 20% of the patients were randomly selected as a test dataset for the final evaluation of the data, and the rest of the patients were used as the training dataset. To find optimal high-level parameters (like the number, size, and type of layers in the networks) and lower-level parameters (like the number of epochs, choice of loss function and activation function, and optimization procedure) in the CNN model, the K-fold cross validation method was used during the training phase [17]. We split the training data into 5 partitions, instantiated 5 identical models, and trained each one on 4 partitions while validating on the remaining partition. The performance of each model was evaluated using the mean squared error (MSE) because of the existence of zero values in the outcome variables. We then computed the average performance over the 5 folds. In the end, the choice of the parameters was a compromise between the model’s performance and computing time, i.e., the model with both the smallest validation error and a shorter computing time was deemed an optimal model. The training, validation, and final evaluation process is shown in Figure S3.

To avoid overuse of the deep learning method for prediction, we also applied a simple multivariate linear regression model as an estimator to predict the 5-year HRQoL scores, and compared the performance between the linear regression model and the CNN model.

### 2.5. Software and Hardware

The descriptive and inferential statistical analyses were performed using Stata 15.1 (StataCorp LLC, College Station, TX, USA). The CNN and multiple linear regression models were achieved using packages scikit-learn 0.21.2 and Keras 2.2.4 in Python 3.6 (Python Software Foundation, https://www.python.org/).

All of the computation was conducted in a computer with a 64-bit Windows 7 Enterprise operation system (Service Pack 1), Intel ® Core TM i5-4210U CPU @ 2.40 GHz, and 16.0 GB random access memory.

## 3. Results

### 3.1. Descriptive Analysis of the Data

In total, 6687 patients registered in SOReg between 2008 and 2012 with complete demographic and preoperative comorbidity information, and preoperative and 5-year HROoL scores were included in the study. The characteristics of the patients are shown in Table 1. Briefly, the average age and BMI of the patients were 42.7 years and 42.3 kg/m2, respectively. More than three quarters (77%) were female and 45% had at least one of the five comorbidities (SAS, hypertension, diabetes, depression, and dyslipidemia) before bariatric surgery.

### 3.2. Performance of the CNN Model in the K-Fold Cross-Validation

We analyzed 11 HRQoL scores in the study. To make our description concise, we used the PF score as an example of our data analysis as follows.

**Figure 1:**
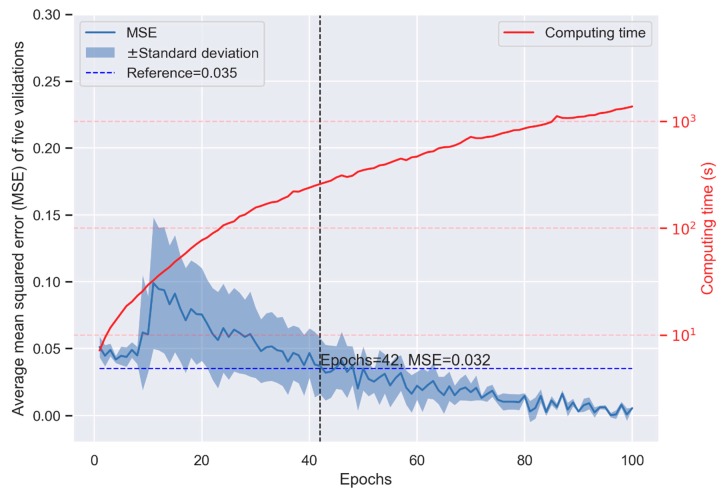
Performance of the convolution neural network (CNN) model in K-fold cross-validation.

**Figure 2:**
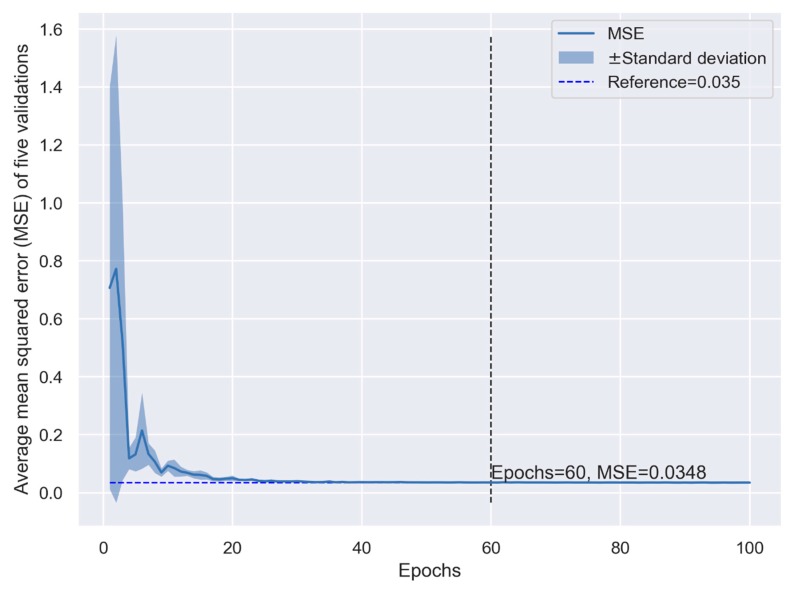
Performance of the simple multivariate linear regression model in K-fold cross-validation.

In general, the performance of the CNN model (measured as the MSE) increased with more convolution layer filters, computation units, and epochs, and decreased with a larger batch size. Although the performance increased with the model’s complexity, the computing time increased exponentially. When we set the number of computation units and filters to be large enough (1000 and 10, respectively) and the batch size was small enough (10), the performance of the CNN model in K-fold cross-validation is shown in Figure 1. The performance was not stable when the number of epochs was small and changed dramatically depending on the random seed used in training (Figure 1). When the number of epochs was >40, the model presented smaller MSE than the linear regression model (0.032 vs. 0.035, Figure 1 and Figure 2). Although more epochs reduced the MSE in the CNN model, the computing time increased exponentially, indicating a higher cost in machine learning (Figure 1). The MSE of the linear regression model appeared constant when the number of epochs >40 (Figure 2), which means the prediction cannot be improved with more epochs. The cross-validation indicates that the CNN model may provide better prediction but at the expense of the computing time.

### 3.3. Performance of the CNN Model in the Final Evaluation

**Figure 3:**
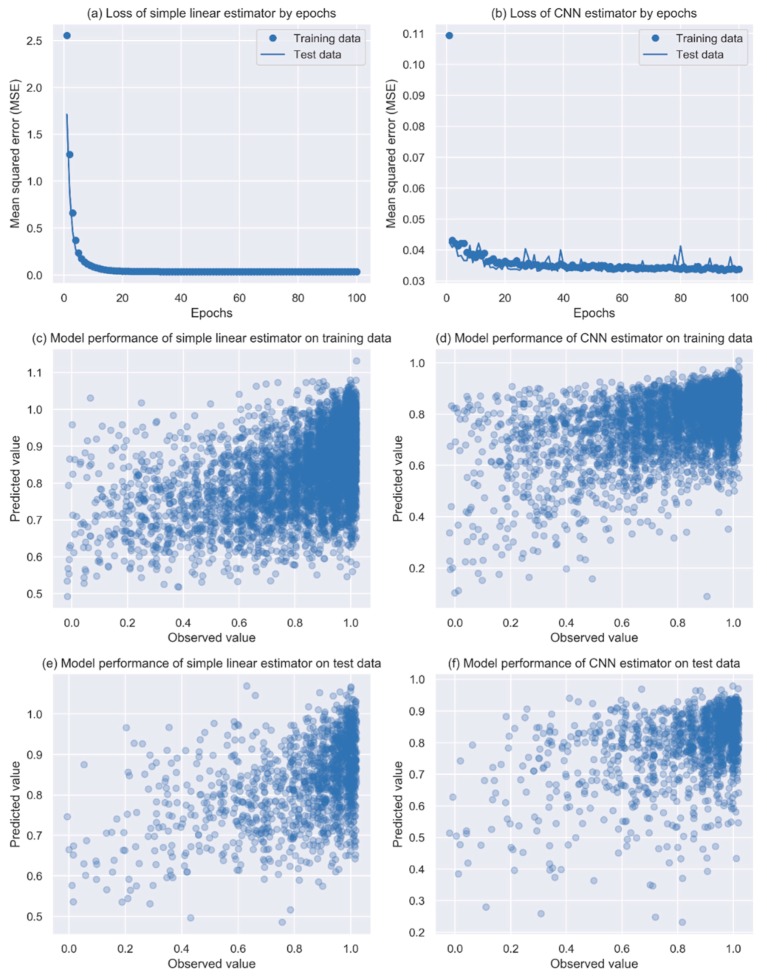
Model performance of the simple linear estimator and the CNN estimator. The dots in the plots (c)–(f) were jittered to avoid a heavy overlap of patients with the same coordinates. CNN, convolution neural network.

When the models were evaluated using the test data that were not seen previously by the models, in general, the CNN model presented a better performance (solid line in Figure 3b) than the linear regression model (solid line in Figure 3a) with epochs >40. Although overfitting was presented sporadically in the CNN model (comparing the solid line with the dotted line in Figure 3b), the performance improved gradually with an increased number of epochs while remaining constant in the linear regression model.

Finally, we used 40 epochs for the CNN model, and predicted PF scores for both the training data and the test data. Clear correlations can be seen between the predicted values and observed values in the training data, with an MSE of 0.032 for the CNN model (Figure 3d and Table 2) compared to the MSE of 0.033 seen in the linear regression model (Figure 3c and Table 2). For the test data, the CNN model had an MSE of 0.035 (Figure 3f and Table 2) compared with 0.034 (Figure 3e and Table 2) from the linear regression model. Although the CNN model provided better prediction than the linear regression model for the test data, the overfitting became apparent in some situations when the model learned patterns more specific to the training data.

### 3.4. Performance of CNN in Predicting Other HRQoL Measures

**Figure 4:**
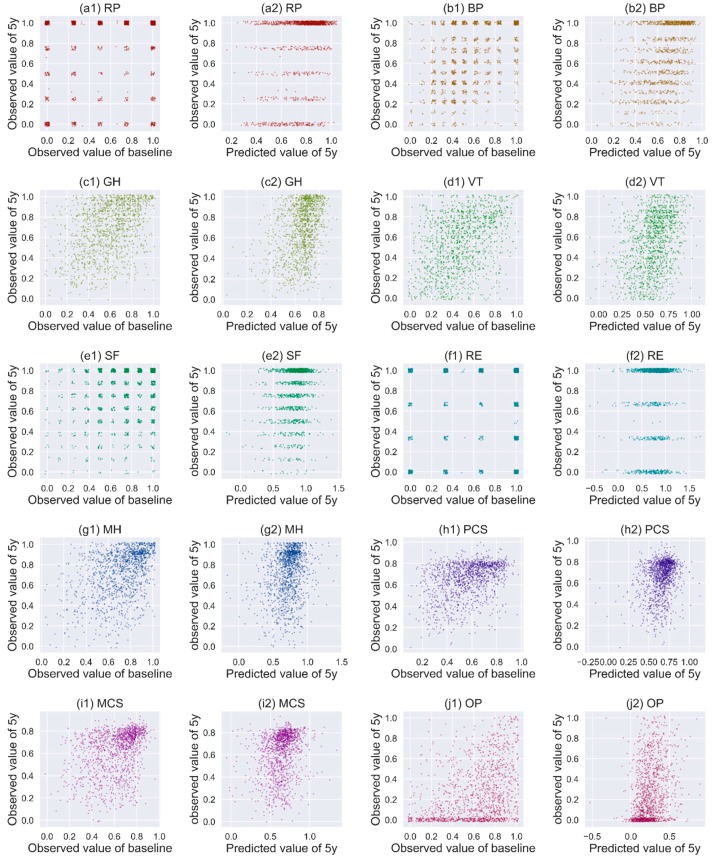
Correlation of the observed 5-year scores with the observed baseline scores and predicted scores for test data. The dots in the plots were jittered to avoid a heavy overlap of patients with the same coordinates. RP, role physical; BP, bodily pain; GH, general health; VT, vitality; SF, social functioning; RE, role emotional; MH, mental health; PCS, summary physical scale; MCS, summary mental scale; OP, obesity-related problems.

The relationships between the baseline and the 5-year scores of other HRQoL measures in the test data are shown in Figure 4. Except for GH and VT, no clear relationship between the baseline and the observed 5-year scores is seen for the HRQoL measures (Figure 4, plots a1–j1). However, the predicted 5-year scores based on the baseline scores and the CNN model show clear correlations with the observed 5-year scores for BP, GH, VT, MH, MCS, and OP (Figure 4, plots a2–j2). 

We compared the performance of the CNN model and the linear regression model for all the HRQoL measures in both the training data and the test data. The CNN model showed an overwhelming advantage in predicting all the HRQoL measures. The MSEs of the CNN model for the training data were 8% to 80% smaller than those of the linear regression model (Table 2). The overfitting was also apparent in the CNN model, i.e., the MSEs of the CNN model for the test data were all greater than those of the linear regression model (Table 2). 

### 3.5. Sensitivity Analysis and Computing Time

We also conducted sensitivity analysis using different scalers and optimizers in data preparation and model compiling, and tuned the hyperparameters using the exhaustive grid search method [18]. Although they showed more or less influence on the models’ performance, the influence was negligible when the number of epochs was large and the batch size was small. The computing time for the CNN model largely depends on the hyperparameter settings of the layers, number of epochs and the batch size for training, and the software and hardware used. In our study, with the model structures and hyperparameters shown in Figure S2, the running time ranged from 70 (epoch = 40, batch size = 10, without cross-validation) to 595 s (epoch = 400, batch size = 10, with five cross-validations) on our computer.

## 4. Discussion

Machine learning methods to predict HRQoL have been used in elderly with chronic diseases [19], cervical cancer patients [20], and osteoarthritis patients [21]. However, to our knowledge, they have not been used to predict the postoperative HRQoL of patients undergoing bariatric surgery. We explored the feasibility and capacity of a deep learning method, i.e., convolution neural network, to predict long-term HRQoL after bariatric surgery using a national register. The study can only be achieved based on a well-maintained and high-quality longitudinal database with long-term follow-up like SOReg [22].

Our results indicate that 5-year HRQoL after bariatric surgery may be well predicted preoperatively for some scale domains like PF, BP, GH, VT, MH, MCS, and OP. In our study, we aimed to evaluate and predict the quality of life of patients after bariatric surgery. Some patients were not “satisfied” even when they lost weight. Other factors, such as complications during follow-up and preoperative pharmacologic drug treatment, are associated with a change of the quality of life after bariatric surgery, whereas age, sex, and preoperative metabolic comorbidity may also play a role [11,23,24,25]. Our findings may provide important information for postoperative care and rehabilitation for this group of patients.

Our research question was about predicting continuous outcomes using supervised deep learning methods, which could be converted to a question of supervised two- or multi-class classification, i.e., to predict whether the quality of life of the patient has improved, remained unchanged, or deteriorated. Although the precision of prediction might be reduced in classification, the accuracy might be enhanced, and the method might be more applicable for clinical use. We would like to investigate the question in future studies.

There has been a warning that healthcare researchers should not be overly enthralled by the promises of deep learning methods [26]. Therefore, to avoid abusing the deep learning method in our study, we also compared the performance of the CNN model with a conventional statistical learning method for continuous variables, using a multivariate linear regression model. Although the conventional statistical methods require sometimes complex processing (feature engineering) to extract the requisite discriminative features, they may provide more interpretable results compared to the deep learning methods. In contrast, the biggest advantage of deep learning methods is that they try to learn high-level features from data in an incremental manner, which eliminates the need for domain expertise and hard-core feature extraction. However, the generalizability of deep learning models relies largely on the data they learned, and overfitting on unseen data is more apparent, as observed in our study. Although there are some ways in which we may reduce overfitting in deep learning models, the rule of thumb is to use more training data.

There are potential limitations to our study. In total, 28,293 patients underwent surgery for a primary gastric bypass between 2008 and 2012 and had a follow-up longer than 5 years when the study was initiated. However, only less than one quarter of the patients who had complete HRQoL information could be used for the machine learning. Compared to the patients who had no or incomplete HRQoL information, the patients with complete relevant data were older (42.7 ± 11.0 vs. 40.4 ± 10.8 years), had fewer males (21.2% vs. 25.1%), and lower BMI (42.3 ± 5.2 vs. 42.8 ± 5.5 kg/m^2^). These factors have already been shown to influence HRQoL [27,28,29]. Because of these systematic differences in HRQoL between the patients with and without HRQoL measures, the generalizability of our CNN model may be questionable. The missing information needs to be imputed in the future for deep machine learning. We would also point out that the CNN built in our study was only based on features from gastric bypass patients, which cannot be generalized to other surgical procedures or health conditions. The application of CNN in predicting prognosis after surgeries still needs to be investigated using large data from the real world.

## 5. Conclusions

CNN can be used to predict long-term HRQoL after bariatric surgery based on the baseline features of patients. The performance of the CNN was found to be better than the traditional multivariate linear regression model; however, its overfitting on unseen data needs to be mitigated by using more features of patients or greater use of training data in the future.

## Figures and Tables

**Table 1:** Characteristics of the patients (*n* = 6687) included in the study, mean (SD) or *n* (%).

	Preoperative		Five Years after Bariatric Surgery	
	Original	Scaled	Original	Scaled
Age (year)	42.7 (11.0)	0.494 (0.197)	47.7 (11.0)	0.494 (0.197)
BMI (kg/m^2^)	42.3 (5.2)	0.241 (0.103)	30.3 (5.2)	0.358 (0.127)
Female	5259 (77%)	NA	5259 (77%)	NA
SAS	680 (10%)	NA	NA	NA
Hypertension	1851 (27%)	NA	NA	NA
Diabetes	990 (15%)	NA	NA	NA
Depression	884 (13%)	NA	NA	NA
Dyslipidemia	747 (11%)	NA	NA	NA
PF	61.6 (21.9)	0.616 (0.219)	84.2 (20.7)	0.842 (0.207)
RP	60.2 (38.9)	0.602 (0.389)	77.8 (36.6)	0.778 (0.366)
BP	56.0 (26.8)	0.560 (0.268)	65.1 (30.8)	0.651 (0.308)
GH	58.2 (21.4)	0.582 (0.214)	68.0 (24.7)	0.680 (0.247)
VT	47.3 (23.0)	0.473 (0.230)	54.5 (26.9)	0.545 (0.269)
SF	74.8 (26.1)	0.748 (0.261)	79.5(26.5)	0.795 (0.265)
RE	75.9 (36.2)	0.759 (0.362)	76.7 (37.9)	0.767 (0.379)
MH	71.5 (19.4)	0.715 (0.194)	72.0 (23.0)	0.720 (0.230)
PCS	38.3 (10.7)	0.567 (0.177)	47.6 (11.1)	0.653 (0.163)
MCS	46.8 (11.7)	0.621 (0.172)	44.6 (13.8)	0.621 (0.192)
OP	61.0 (26.3)	0.610 (0.263)	25.6 (27.4)	0.256 (0.274)

SD, standard deviation; NA, not applicable; BMI, body mass index; SAS, sleep apnea syndrome; PF, physical functioning; RP, role-physical; BP, bodily pain; GH, general health; VT, vitality; SF, social functioning; RE, role-emotional; MH, mental health; PCS, summary physical scale; MCS, summary mental scale; OP, obesity-related problems.

**Table 2:** Mean squared errors (MSEs) of the CNN model and the multivariate linear regression model.

HRQoL Measure	Training Data		Test Data	
	CNN Model	Linear Regression Model	CNN Model	Linear Regression Model
PF	0.0316	0.0329	0.0350	0.0343
RP	0.1078	0.1178	0.1324	0.1211
BP	0.0604	0.0763	0.0898	0.0772
GH	0.0280	0.0497	0.0618	0.0508
VT	0.0303	0.0572	0.0914	0.0625
SF	0.0213	0.0600	0.0995	0.0588
RE	0.0393	0.1275	0.2118	0.1269
MH	0.0119	0.0427	0.0807	0.0416
PCS	0.0087	0.0210	0.0333	0.0219
MCS	0.0075	0.0301	0.0584	0.0305
OP	0.0450	0.0625	0.0750	0.0608

PF, physical functioning; RP, role physical; BP, bodily pain; GH, general health; VT, vitality; SF, social functioning; RE, role emotional; MH, mental health; PCS, summary physical scale; MCS, summary mental scale; OP, obesity-related problems.

## Supplementary Materials

The following are available online at https://www.mdpi.com/2077-0383/8/12/2149/s1, Figure S1: Physical functioning (PF) scores before and after bariatric surgery of 6687 patients used in the study. Figure S2: Structure of the conventional neural network (CNN) model used in the study. Figure S3: Process of training, validation and evaluation
