# Supplementary material for: Predicting Long-Term Health-Related Quality of Life after Bariatric Surgery Using a Conventional Neural Network: A Study Based on the Scandinavian Obesity Surgery Registry
Source: J Clin Med. 2019 Dec 5;8(12):2149. doi: 10.3390/jcm8122149 (PMC6947423; doi:10.3390/jcm8122149)
Supplement: Supplementary file 1 [file jcm-08-02149-s001.pdf]

Supplemental materials

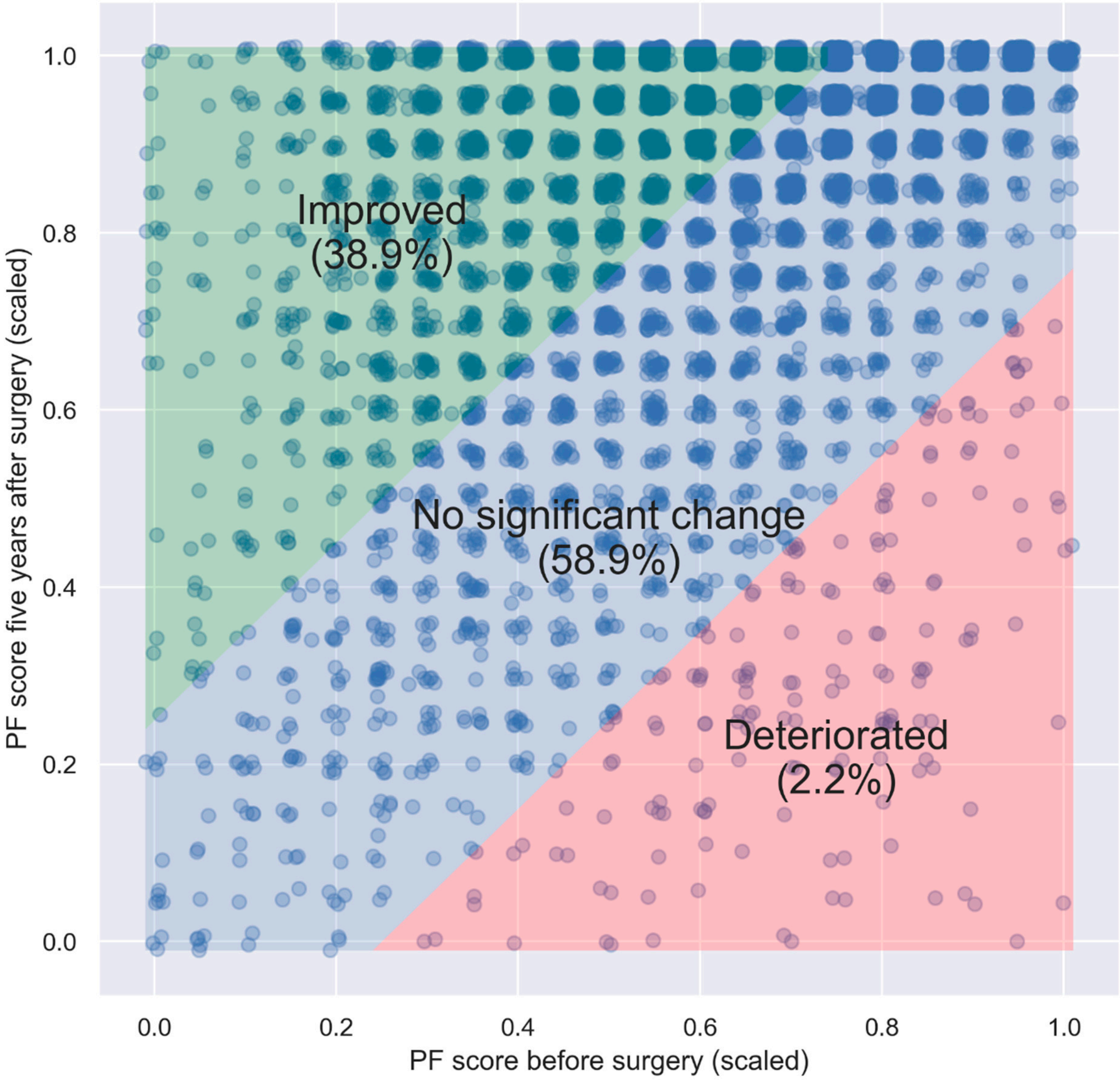

Figure S1. Physical functioning (PF) scores before and after bariatric surgery of 6687 patients used in the study. The dots in the figure are jittered to avoid heavy overlap of patients with the same coordinates.

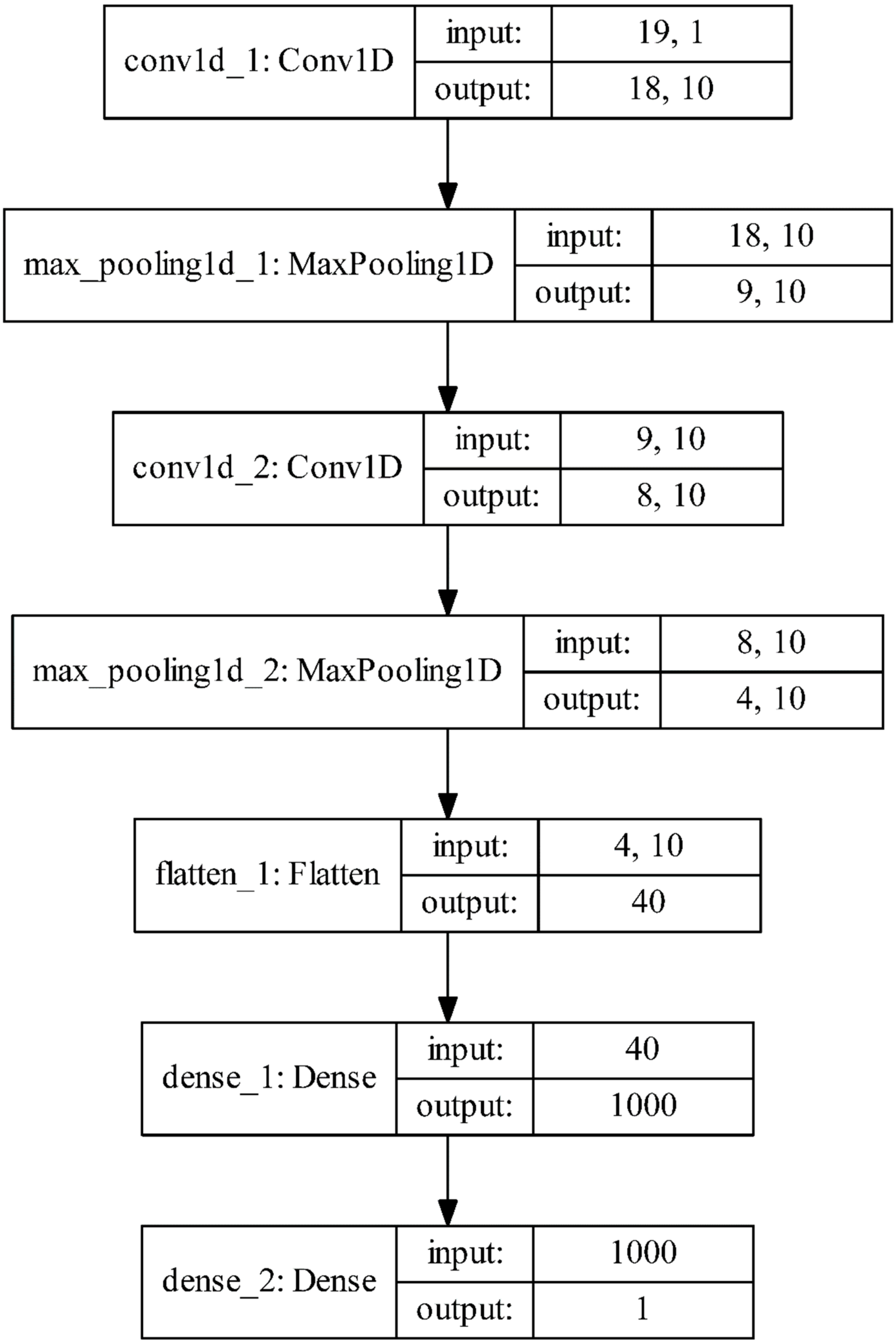

Figure S2. Structure of the conventional neural network (CNN) model used in the study.

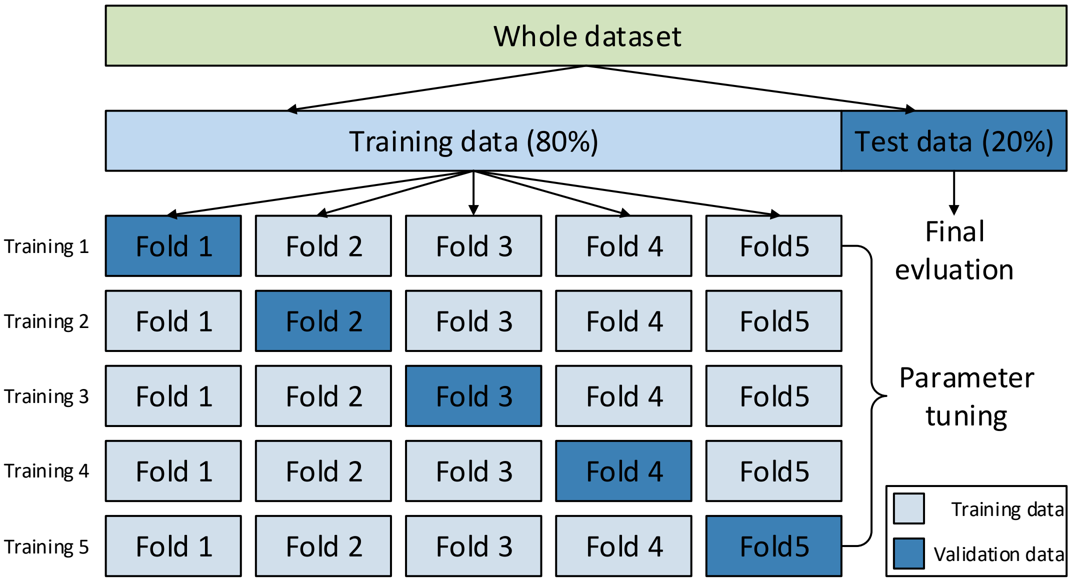

Figure S3. Process of training, validation and evaluation.
